# Giant two-photon upconversion from 2D exciton in doubly-resonant plasmonic nanocavity

**DOI:** 10.1038/s41377-025-02010-w

**Published:** 2025-09-10

**Authors:** Fangxun Liu, Haiyi Liu, Cheng Chi, Wenqi Qian, Yuchen Dai, Guangyi Tao, Sihan Lin, Shihan Ding, Menghan Yu, Hongliang Liu, Lie Lin, Pengfei Qi, Zheyu Fang, Weiwei Liu

**Affiliations:** 1 Institute of Modern Optics, Nankai University, Tianjin Key Laboratory of Micro-scale Optical Information Science and Technology, Tianjin, China; 2 Beijing Engineering Research Center of Mixed Reality and Advanced Display, School of Optics and Photonics, Beijing Institute of Technology, Beijing, China; 3 School of Physics, State Key Laboratory for Mesoscopic Physics, Academy for Advanced Interdisciplinary Studies, Collaborative Innovation Center of Quantum Matter, Nano-optoelectronics Frontier Center of Ministry of Education, Peking University, Beijing, China; 4 Academy for Advanced Interdisciplinary Studies, Nankai University, Tianjin, China

**Keywords:** Ultrafast photonics, Nonlinear optics

## Abstract

Photon upconversion through high harmonic generation, multiphoton absorption, Auger recombination and phonon scattering performs a vital role in energy conversion and renormalization. Considering the reduced dielectric screening and enhanced Coulomb interactions, semiconductor monolayers provide a promising platform to explore photon upconversion at room temperature. Additionally, two-photon upconversion was recently demonstrated as an emerging technique to probe the excitonic dark states due to the extraordinary selection rule compared with conventional excitation. However, highly efficient two-photon upconversion still remains challenging due to the limited multiphoton absorption efficiency and long radiative lifetimes. Here, a 2440-fold enhancement of two-photon luminescence (TPL) is achieved in doubly resonant plasmonic nanocavities due to the amplified light collection, enhanced excitation rate, and increased quantum efficiency. To gain more insight into the attractive doubly resonant enhancement in such a plasmon−exciton coupling system, the intriguing thermally tuned excitonic upconversion and optimized amplification factor >3000 are realized at 350 K. Meanwhile, the single resonance enhanced photoluminescence (PL) (~890-fold) and second-harmonic generation (SHG) (~134-fold) are elaborately demonstrated. These results establish a foundation for developing cost-effective, high-performance nonlinear photonic devices and probing fine excitonic states via configuring plasmonic nanocavities.

## Abstract (web summary)

Two-photon upconversion of 2D exciton was enhanced by 2440-fold in doubly-resonant nanocavity due to combined field localization and Purcell effect.

## Graphical abstract

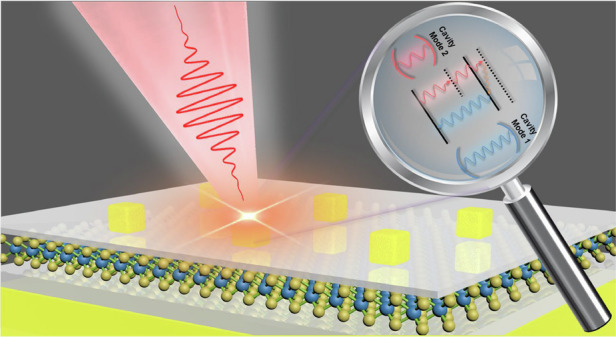

## Introduction

Excitons, the hydrogen-like bosonic quasiparticles formed by electron−hole pairs through Coulomb attraction, often dominate the optical and electrical properties of most semiconductor materials^1,2^. Transition metal dichalcogenides (TMDCs) monolayers, as an important class of two-dimensional (2D) semiconductors, provide a platform of enormous potential to explore exciton physics and applications at room temperature by virtue of the reduced dielectric screening, enhanced Coulomb interactions and relatively large effective masses of charge carriers^3–6^. Additionally, valley pseudospin has been demonstrated as an alternative steerable degree of freedom due to the time-reversal symmetry, broken inversion symmetry, and strong spin–orbit splitting in monolayer TMDCs^7–11^. Therefore, 2D excitons in TMDCs monolayers have attracted great interest to realize the interesting exciton behaviors (Hall effects, gas-liquid transition, Bose-Einstein Condensation, etc^12–14^) and the promising excitonic devices (modulators, switches, storage devices, field-gradient devices, transistors, etc^15–20^).

Photon upconversion is an anti-Stokes process to emit a photon at energy higher than excitation photon energy through molecular triplet–triplet annihilation, second harmonic generation or multiphoton absorption processes^21–25^. The efficient upconversion photoemission has been extensively investigated in various luminescent systems such as organic dyes, quantum dots, nanobelts, carbon nanotubes and lanthanide-doped upconversion nanoparticles^26–30^. Recently, the upconversion photoemission with larger energy gain of 30–150 meV in monolayer TMDCs at cryogenic and room temperatures has been reported through phonon-mediated upconversion^31–35^, in which the larger energy gain is severely limited by the phonon energy of tens or hundreds of millielectron volts. The remarkable multiphoton absorption of monolayer TMDCs opens up new avenues to break the limitation^36^. Furthermore, the selection rule of transition depends on the symmetry of the final state for excitonic Rydberg-like states with definite parity, similar to the hydrogen model: one-photon transitions can only reach excitonic states with even parity, while two-photon transitions reach states with odd parity. Consequently, the two-photon upconversion is also an indispensable technique to probe the excitonic dark states that are absent in the linear optical spectrum^37^. Nevertheless, direct realization of giant upconversion efficiency at low-threshold excitation intensity for 2D excitons still remains challenging owing to the limited multiphoton absorption efficiency and long radiative lifetimes.

As is well known, coupling the photon emitters to an optical cavity can significantly change the interaction between the emitter and its local optical environment^38,39^. By integrating monolayer TMDCs into a plasmonic nanocavity, (a) the deep subwavelength mode volume of the cavity resonances, i.e., the localized surface plasmons (LSPs) can provide locally enhanced electromagnetic fields, thereby enhancing the multiphoton absorption of the monolayer TMDCs, (b) the spontaneous emission rate of the emitter can be accelerated via Purcell factor in weak coupling regime, leading to a phenomenon known as plasmon-enhanced fluorescence^40–42^. Moreover, the resonance frequencies of LSPs can be conveniently tailored by changing the size, shape, and interparticle separation. Therefore, the plasmonic nanocavities are promising to realize giant two-photon upconverted PL in monolayer TMDCs, by enhancing multiphoton absorption and shortening spontaneous radiative lifetimes.

In this work, by employing an elaborate doubly resonant plasmonic nanocavity, we observe an unprecedented 2440-fold amplification in the two-photon upconverted emission from 2D excitons, which can be attributed to the elevated excitation rate, magnified light collection, and quantum efficiency amplification arising from the Purcell effect. To gain more insight into the attractive doubly resonant enhancement in such a plasmon−exciton coupling system, the intriguing thermally tuned excitonic upconversion and optimized amplification factor >3000 are realized at 350 K. Meanwhile, the single resonance enhanced PL (~890-fold) and SHG (~134-fold) are elaborately demonstrated. These findings establish a foundation for developing cost-effective, high-performance nonlinear photonic devices and probing fine excitonic states via configuring plasmonic nanocavities.

## Results

### Design and characterization of plasmonic upconversion devices

Two-photon absorption (TPA)-driven PL upconversion generally suffers from low conversion efficiency, thereby choosing the monolayer with higher PL quantum yield will facilitate the observation of TPL of 2D excitons. Monolayer WS_2_ was selected for this study owing to its direct bandgap of 2.0 eV (620 nm), which enables efficient TPL when excited by near-infrared laser pulses. Furthermore, monolayer WS_2_ exhibits a superior PL quantum yield (≈6%) compared with other 2D semiconductors like monolayer MoS₂ (≈0.1%)^43^. Atomic force microscopy (AFM) characterization confirms the monolayer nature of WS_2_ flakes, with measured thickness of ∼0.9 nm (see Supplementary Information S1 for more details). Besides, based on the PL and Raman spectroscopy (see Supplementary Information S2 for more details), we have confirmed that the used WS_2_ is a high-quality monolayer material with low defect density. Additionally, the 800 nm laser was chosen for TPL measurements due to its commercial availability as a well-established Ti:sapphire laser source, along with resonance matching and effective TPA in WS_2_. Moreover, the particle-on-mirror plasmonic nanocavity architecture, consisting of a nanoparticle coupled to an ultrasmooth metallic film, has recently emerged as a versatile and cost-effective platform for optical nonlinearity enhancement, offering both superior performance and fabrication scalability^44,45^. Optical excitation generates surface plasmons in the nanogap between the metallic nanoparticles and underlying film, creating intense near-field enhancement^46,47^. The nanocavity’s resonance wavelength can be adjusted by controlling the spacer thickness and the nanoparticle size. Before fabrication, three-dimensional finite-difference time-domain (3D-FDTD) simulations were performed to optimize the nanocavity geometry, ensuring spectral matching between plasmonic resonances and the characteristic excitation/emission bands of 2D excitons. (see Supplementary Information Fig. S3 for more details).

**Fig. 1:**
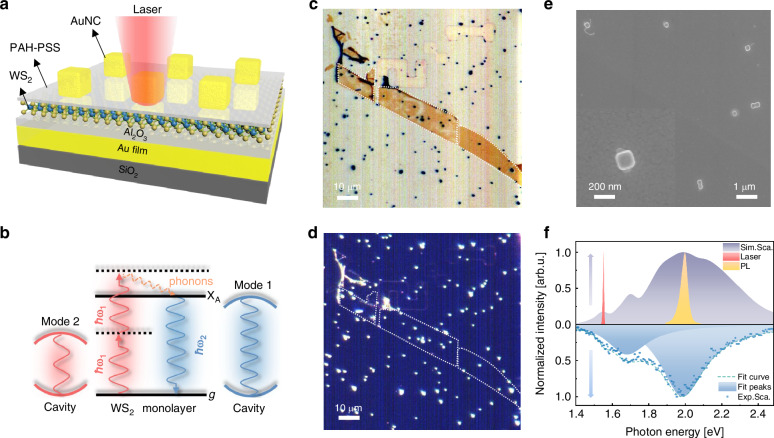
**Design and characterization of plasmonic upconversion devices**. **a** Schematic of the designed AuNCs/WS_2_/substrate plasmonic upconverter devices. **b** Desired band alignment diagram of monolayer WS_2_ in plasmonic nanocavities and the photon upconversion process of 2D excitons. **c** Bright- and **d** dark-field microscope optical images of a representative sample. **e** Scanning electron micrograph of 170 nm AuNCs on the substrate, and the inset shows a magnified image from the top view. **f** Excitation laser and monolayer WS_2_ PL spectra overlapping the simulated scattering spectrum of a plasmonic nanocavity, which is consistent with experimental results. The excitation laser and upconverted emission spectra are doubly resonant with plasmonic cavity modes at 1.55 and 2.0 eV, respectively. All the spectra are normalized

To simultaneously enhance upconverted excitation and emission processes in doubly resonant plasmonic nanocavities, mechanically exfoliated monolayer WS_2_ is inserted into the gaps between nanoparticles and a mirror underneath^38^. Figure 1a presents the designed device architecture of the Au nanocubes (AuNCs)/WS_2_/substrate plasmonic upconverter, featuring a layered substrate composed of a 7 nm Al_2_O_3_ spacer and a 50 nm Au film evaporated on a Si/SiO_2_ wafer. Figure 1b presents the band alignment diagram for WS_2_ within the plasmonic nanocavity, illustrating the TPA upconversion mechanism. Simultaneous absorption of two sub-bandgap photons excites valence band electrons to a virtual intermediate state, followed by relaxation to the conduction band minimum (CBM) primarily via multiphonon relaxation^48–50^. As the energy difference between the virtual intermediate state and the CBM is significantly higher than WS_2_ phonon energy around room temperature, multiple phonons are required for this relaxation. The recombination of excited electrons at the conduction band minimum with holes in the valence band produces photons at energies matching the WS_2_ bandgap. A black dashed line indicates the virtual intermediate state, while red and blue wavy arrows denote incident and emitted photons, respectively ($\hslash {\omega }_{2} > \hslash {\omega }_{1}$). The orange waveform denotes intraband relaxation processes. In our optimized plasmonic nanocavity design, dual resonance conditions—simultaneously matching excitation (1.55 eV) and emission (2.0 eV) energies—collectively enhance all steps of the upconversion process, yielding significantly improved conversion efficiency.

Figure 1c and d show bright- and dark-field micrographs of the representative device, respectively. The exfoliated monolayer WS_2_ was transferred onto an Al_2_O_3_(7 nm)/Au (50 nm)/SiO_2_/Si substrate fabricated by evaporation coating. AuNCs with an average edge length of 170 nm (Fig. 1e), synthesized via seed-mediated growth, were sparsely deposited onto WS_2_ monolayers by controlled drop-casting. A nanoscale organic spacer layer [poly(allylamine) hydrochloride (PAH)/poly(sodium-pstyrenesulfonate) (PSS), ~1 nm] was incorporated between the WS_2_ and AuNCs to prevent hot carrier injection from LSPs or surface plasmon polaritons (SPPs) into the semiconductor.

Figure. 1f compares simulated and experimental scattering spectra, demonstrating spectral alignment between 800 nm excitation laser, 620 nm upconversion emission, and plasmonic cavity modes (see Supplementary Information S4 for more details). The excellent agreement between simulation and experiment validates our nanocavity design. Both the excitation laser (1.55 eV) and WS_2_ PL (peak at 2.0 eV) spectrally overlap with the nanocavity’s scattering spectrum, establishing dual-mode plasmonic resonance at both (i) the pump energy (1.55 eV mode) and (ii) emission energy (2.0 eV mode). The observed 450 meV energy difference (Δ*E*) significantly exceeds the characteristic phonon energy in WS_2_ (~30 meV), confirming a TPA mechanism rather than phonon-assisted upconversion. Note that TPA and TPL have been extensively studied in past decades^51,52^, and the rigorous theory of multiphoton-assisted absorption and emission in semiconductors was developed in ref. ^53^.

### Amplified excitonic upconverted emission by plasmonic cavity

**Fig. 2:**
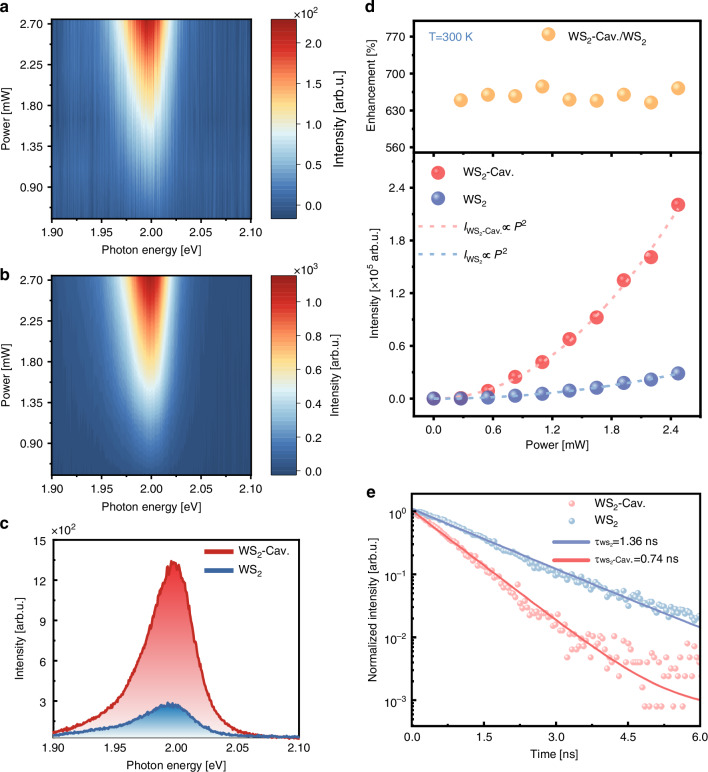
**Amplified excitonic upconverted emission by plasmonic cavity**. **a** Excitation power-dependent upconverted emission spectra for monolayer WS_2_ on Au/SiO_2_/Si. **b** Excitation power-dependent upconverted emission spectra for monolayer WS_2_ in the designed plasmonic cavity. **c** Enhanced upconverted emission spectra of monolayer WS_2_ in the designed plasmonic cavity at an excitation power of 2.75 mW. **d** Excitation power-dependent integrated upconverted emission intensity (down) and enhancement (top) for monolayer WS_2_ in the designed plasmonic cavity. The experimental results (scattering points) can be fitted well by the blue and red dotted lines based on the power law formula *I* = b*P*^*α*^. **e** Comparison of the normalized time-resolved luminescence decay for WS_2_ deposited on the Au film (blue) and WS_2_ enhanced by the nanocavity mode (red) at an emission wavelength of 620 nm

To systematically investigate plasmon-enhanced upconversion, we conducted excitation power-dependent measurements using 1.55 eV photons under ambient conditions (T = 300 K, 1 atm). Firstly, experiments conducted on monolayer WS_2_ transferred to Au/SiO_2_/Si substrates revealed an increase in upconverted emission intensity with excitation power (Fig. 2a). The invariant spectral lineshape and peak position across the measured power range confirm negligible bandgap renormalization or lattice heating effects. However, above a threshold power of 3.3 mW, the PL peak exhibits a progressive red-shift of approximately 20 meV as the power increases to 8.8 mW, owing to laser-induced heating that exceeds the thermal dissipation capacity of monolayer WS_2_ (see Supplementary Information Fig. S5 for more details). While the spectral lineshape remained unchanged, high-power excitation induced both a progressive red-shift and linewidth broadening^49,54,55^. Consequently, all subsequent measurements were limited to excitation powers under 3.3 mW to prevent possible thermal or damaging effects.

In our optimized plasmonic upconverter (Fig. 1a and f), dual resonance conditions simultaneously enhance excitation and upconverted emission processes. Comparative measurements at 2.75 mW excitation power reveal a 6.7-fold intensity enhancement (Fig. 2c) for cavity-coupled WS_2_ relative to the monolayer WS_2_ on Au/SiO_2_/Si reference (see Supplementary Information S6 for more plasmonic nanocavities), confirming the predicted plasmonic amplification. To quantitatively characterize the power dependence of plasmonic enhancement, we systematically mapped the upconverted emission from cavity-coupled WS_2_ varying excitation power, as presented in Fig. 2b. The spectral stability observed in cavity-coupled WS_2_ compared to Fig. 2a confirms the efficacy of the Al_2_O_3_/PAH-PSS spacer in blocking hot electron injection from LSPs of SPPs. Critically, the absence of trion formation (no emergent peak at ~1.97 eV) demonstrates successful suppression of charge transfer despite strong plasmon-exciton coupling. Additionally, based on the PL and Raman spectroscopy (see Supplementary Information S2 for more details), we have confirmed that there is no significant strain effect in our designed plasmonic nanocavity to influence the emission enhancement.

To elucidate the underlying physical mechanisms governing emission enhancement in the Au-WS_2_ hybrid structure, the dependence of WS_2_ on Au/SiO_2_/Si and the plasmonic cavity signal intensities on excitation laser power were plotted in Fig. 2d. The superlinearly increased upconversion intensities can be well fitted by *I* = b*P*^*α*^, where *I* and *P* are the integrated PL intensity and laser power, respectively. The exponent *α* = 2 for both Au/SiO_2_/Si and the plasmonic cavity confirms that here the upconversion mechanism can be attributed to coherent TPA rather than phonon assisted upconversion characterized by one-photon excitation which features a linear or sublinear power dependence^56–58^.

Given the highly localized nature of plasmonic enhancement, the actual upconversion enhancement within these hotspot regions significantly exceeds the spatially averaged values reported in Fig. 2d. Commonly, to quantify the actual upconversion enhancement in plasmonic cavities, the PL enhancement factor can be defined as^41^

1 $$\langle EF\rangle =\frac{{I}_{PC}-{I}_{0}}{{I}_{0}}\frac{{S}_{0}}{{S}_{PC}}$$

where *I*_*PC*_ and *I*_0_ are the total upconversion intensity of cavity-coupled and solely monolayer WS_2_, respectively. *S*_0_ denotes the excitation area (10.45 μm^2^, see Supplementary Information S7 for more details), and *S*_PC_ represents the cavity hotspot area enhancing the upconversion. We assume that *S*_PC_ is the area contacting the monolayer WS_2_ with the plasmonic cavity (0.029 μm^2^). An enhancement range of 2330–2440-fold can be achieved, corresponding to Fig. 2d. The absence of significant power dependence in the enhancement factor indicates that the upconversion process remains unaffected by saturation effects across the investigated power range.

Subsequently, we characterized the excited-state decay dynamics through time-resolved PL spectroscopy (Fig. 2e), comparing WS_2_ on Au films versus nanocavity-embedded samples (see Supplementary Information S8 for more details). Normalized transient kinetic traces at the emission wavelength (620 nm) were obtained. The WS_2_ deposited on the gold film exhibited monoexponential decay, with a relatively slow decay lifetime of 1.36 ns at a wavelength of 620 nm, while the nanocavity-coupled WS_2_ demonstrated a significantly reduced luminescence lifetime of 0.74 ns. The physical mechanism of this phenomenon focuses on modification of the emitter environment to enhance the spontaneous emission rate^59^, known as the Purcell effect^60^. In next section, 3D-FDTD simulations were performed to gain more insight into the Purcell-enhanced upconversion process, which offers a detailed analysis and explanation.

### Physical scenario of enhanced upconversion in plasmonic cavity

**Fig. 3:**
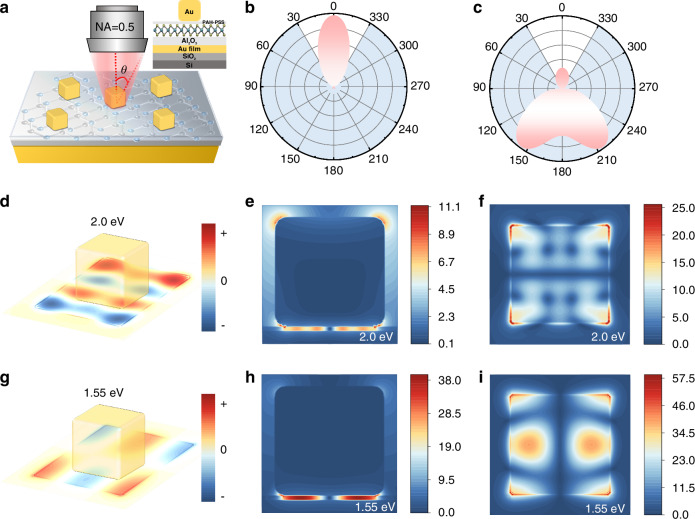
**Physical scenario of enhanced upconversion in plasmonic cavity**. **a** Schematic of the setup for collecting upconverted emissions in our experiments. The inset presents a cross-sectional diagram of the hybrid nanocavity. **b,** **c** Far-field angular radiation patterns for monolayer WS_2_ in the plasmonic cavity (**b**) and free space (**c**). **d**–**f** Charge and field distribution (|*E*/E_0_ | ) around the plasmonic cavity for the mode at the emitted photon energy (2.0 eV). **g**–**i** Charge and field distribution (|*E*/E_0_ | ) around the plasmonic cavity for the mode at excitation photon energy (1.55 eV)

The substantial enhancement of upconverted emission in doubly resonant plasmonic nanocavities stems from three synergistic mechanisms: improved light collection efficiency, increased excitation rates, and enhanced quantum efficiency due to the Purcell effect. Collectively, these effects yield an average upconversion enhancement of 2330–2440-fold. The plasmonic nanocavity can be regarded as a nanoscale patch antenna, effectively improving emission directionality to optimize light collection in optical systems with a fixed numerical aperture (NA) (Fig. 3a). 3D-FDTD simulations reveal the antenna’s radiation characteristics (see Supplementary Information Fig. S9 and S10 for more details). Fig. 3b demonstrates that an in-plane dipole source (620 nm center wavelength, 25 nm spectral width) matching WS_2_ monolayer PL produces a far-field pattern featuring a surface-normal-oriented single lobe. The objective lens (NA = 0.5) collects 59.1% of emitted light, representing a 1.6-fold improvement over monolayer WS_2_ on Au/SiO_2_/Si (Fig. 3c).

To further elucidate the plasmonic cavity’s light-field modulation and upconversion enhancement mechanisms, 3D-FDTD simulations of its charge and field distributions were conducted. Within the plasmonic cavity, AuNCs interact with underlying Au film, generating image dipoles that couple with SPPs, to produce strong field enhancement in both in-plane and out-of-plane components (see Supplementary Information S11 for more details). Figure 3d and g show the charge distributions at the monolayer WS_2_, corresponding to resonance of 2.0 and 1.55 eV in the far-field scattering spectrum (Fig. 1f), respectively. The results indicate that the 2.0 eV resonance originates from mixed dipolar modes, while the 1.55 eV resonance stems from a coupled quadrupolar mode. Figure 3e and h display the xz-plane field distributions for both plasmonic cavity modes, while Fig. 3f and i present the corresponding normalized field enhancements (|*E*/E₀|) within the WS_2_ monolayer (see Supplementary Information Fig. S12 for more details). These results demonstrate strong light confinement in the nanocavity gap, with maximum field enhancements reaching |*E*/E₀| = 58 at 1.55 eV resonance.

Furthermore, the observed 25-fold field enhancement at the A-exciton PL energy (2.0 eV) reveals a significant Purcell effect, generating field localization not only at the AuNC-WS_2_ interface corners, but also extending to the upper rounded corners of the nanocube-air interface, creating localized hot spots. The enhancement of spontaneous upconversion emission rate can be attributed to Purcell-effect-mediated manipulation of the nanoparticle’s local density of states (LDOS). Fermi’s golden rule establishes that the emission rate (*γ*_sp_) for an emitter in a confined environment scales with the LDOS at the emission frequency:

2 $${\gamma }_{sp}(r)=\frac{\pi \omega }{3\hslash {\varepsilon }_{0}}{|p|}^{2}\rho (r,\omega )$$

where *ω* is the emission frequency, *p* denotes the emitter transition dipole moment, *r* is the position and *ρ*(*r*, *ω*) is the LDOS. Within a high-quality resonant cavity, the LDOS is significantly enhanced, leading to amplification of spontaneous emission rates for embedded emitters by Purcell factor^61^:

3 $$F=\frac{{\gamma }_{sp}}{{\gamma }_{0}}=\frac{4}{4{\pi }^{2}}\left(\frac{Q}{{V}_{\mathrm{mode}}}\right){\left(\frac{\lambda }{n}\right)}^{3}$$

where *γ*_0_ is the spontaneous emission rate in free space, respectively, *Q* is the quality factor, *V*_mode_ is the mode volume, *λ* is the resonant wavelength, and *n* is the refractive index. The formula reveals that efficient emission rate enhancement demands strong spatial confinement; hence, the plasmonic nanocavities achieve remarkable Purcell enhancement primarily through nanoscopic mode volumes despite their relatively moderate quality factors^38,47^. Eventually, 2D excitons in the optimized plasmonic cavity demonstrate dramatic enhancement of both spontaneous emission rates and luminescence intensity.

### Thermal tuning upconversion for plasmon−exciton coupling system

**Fig. 4:**
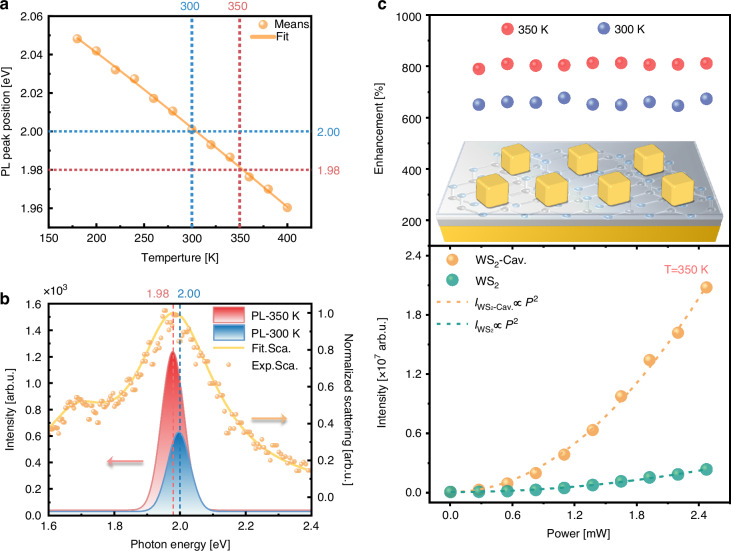
**Thermal tuning upconversion for plasmon−exciton coupling system**. **a** Temperature-dependent PL peak position for WS_2_ on Au/SiO_2_/Si. Yellow lines: fitted results by Varshni equation. **b** Scattering spectrum (yellow dots) of the hybrid system and PL spectrum of the pristine monolayer WS_2_ at 350 K (red curve) and 300 K (blue curve) on the Au/SiO_2_/Si substrate. The measured scattering spectrum is fitted with Gaussian functions (yellow curve). **c** Excitation power-dependent integrated upconverted emission intensity (down) and enhancement (top) for monolayer WS_2_ in the designed plasmonic cavity. The experimental results (scattering points) can be fitted well by the green and yellow dotted lines based on the power law formula *I* = b*P*^*α*^. In the illustration above, red (blue) dots represent the enhancement factor at 350 K (300 K). The inset is a schematic diagram of the plasmonic cavity structure

To explore exciton energy dependent upconversion, the evolution of the coupled system for different temperatures was investigated. The WS_2_ exciton is temperature-dependent (see Supplementary Information Fig. S13 for more details)^62^, while the resonance energies of the plasmonic nanocavities show negligible thermal shift^63,64^. In accordance with ref. ^62^, here the measured PL peak also reveals strong temperature dependencies. The change in exciton peak position of WS_2_ on Au/SiO_2_/Si with temperature increasing from 180 to 400 K is plotted in Fig. 4a. The temperature evolution of PL peak positions typically follows the empirical Varshni relation^65^:

4 $${E}_{g}(T)={E}_{g}(0)-\alpha \frac{{T}^{2}}{T+\beta }$$

where ${E}_{g}(0)$ represents the band gap at *T* = 0 K, $\beta$ is a constant close to the Debye temperature of the material, and $\alpha$ is the bandgap energy temperature coefficient. Figure 4a displays the temperature dependence of the PL peak position, with the dashed line representing the theoretical fit using Eq. (4). The good agreement between the formula and experimental data confirms the validity of the phenomenological description. The fitting parameters are $\alpha =$(4.43 ± 0.08) × 10^−4 ^eV K^−1^ and $\beta =$90 ± 8 K. The blue and red dashed lines in Fig. 4a indicate the PL peak positions of WS_2_ on Au/SiO_2_/Si at 300 K and 350 K, respectively. Such a strong thermal sensitivity of A-excitons enables active tuning of plasmon-exciton coupling in WS_2_-nanocavity hybrid systems.

The blue and red curves of Fig. 4b manifest the PL spectra of pristine monolayer WS_2_ on Au/SiO_2_/Si at 300 K and 350 K, respectively. Dark-field scattering spectrum of the heterostructure is also included for reference (yellow dots), which is fitted with multi-Gaussian functions. As the temperature of the 2D exciton-plasmonic nanocavity coupling system increases from 300 K to 350 K, the upconverted emission spectra increasingly overlap with the characteristic peak in the experimental scattering spectrum of the plasmonic nanocavity, thereby implying that the coupling system at 350 K has greater enhancement potential. The experimental results, as depicted in Fig. 4c, illustrate that the enhancement factor of two-photon upconversion PL has further increased by 25% relative to room temperature, which is in accordance with expectations. Combined with the aforementioned numerical analysis, the 〈EF〉 was estimated to exceed 3000 for gap plasmon-coupled WS_2_ at 350 K relative to WS_2_ on Au film, illustrating the significant sensitivity of 〈EF〉 to the spectra overlap between linear scattering and TPL spectrum. Overall, thermally tuned excitonic upconversion for the plasmon−exciton coupling system was demonstrated.

### Enhanced PL and SHG in nanocavity by single resonance effect

**Fig. 5:**
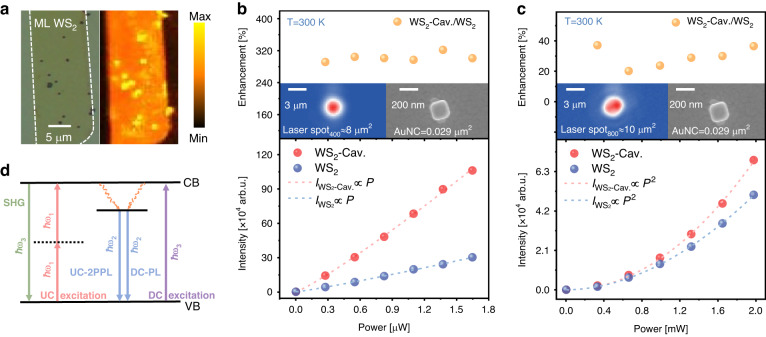
**Enhanced PL and SHG in nanocavity by single resonance effect**. **a** PL maps of the plasmonic nanocavity effect on exciton emission excited by the photon energy of 2.33 eV. **b** (downconversion PL), **c**(SHG) Excitation power-dependent integrated upconverted emission intensity (down) and enhancement (top) for monolayer WS_2_ in the designed plasmonic cavity. The experimental results can be fitted well by the blue and red dotted lines based on the power law formula *I* = b*P*^*α*^. The EMCCD image of the laser spot and the SEM image of the Au nanoparticle are shown as insets. **d** Schematic of downconversion, two-photon excited fluorescence and SHG processes, where UC and DC correspond to the upconversion and downconversion processes

To deepen our understanding on the giant enhancement of TPL in the doubly resonant plasmonic cavity, the enhanced PL and SHG were investigated in the same cavity corresponding to single resonances. In contrast to the upconversion, the conventional PL, namely downconversion, describes the general Stokes process in semiconductors, resulting in reduced energy of emitted photon compared with absorbed one. The conventional PL maps of the upconverter device under the excitation photon energy of 2.33 eV are illustrated in Fig. 5a, where the plasmonic nanocavity can be clearly identified from the monolayer WS_2_ due to the PL efficiency difference. Due to the long-range diffusion of excitons, focal spot size, and instrument response function, the enhanced luminescence does not localize at the nanocube scale. Note that here the conventional PL is enhanced only by the Purcell effect induced by cavity mode at 2.0 eV, it can be expected that the enhancement factor of the PL map by the nanocavities is much smaller than that of the doubly resonant upconversion. For clarity, the power dependence of excitonic downconversion excited at 3.1 eV was measured, as illustrated in Fig. 5b (see Supplementary Information Fig. S14 for more details). It was further corroborated that the enhancement of the downconversion PL is as large as 3.2-fold. Considering the excitation laser spot (8.04 μm^2^, see Supplementary Information S7 for more details) relative to the nanocube size, the effective enhancement factor of 890-fold was achieved.

To further examine the enhancement effect of the other cavity mode at 1.55 eV, the effective enhancement factor of SHG for fundamental wave with photon energy of 1.55 eV was then measured and estimated. The SHG can be confirmed by the quadratic dependence on the excitation laser power, as shown in Fig. 5c (see Supplementary Information Fig. S15 for more details). Here cavity mode at 1.55 eV is resonant with the fundamental wave. In principle, the generated SHG signals can be efficiently enhanced by localized field of fundamental wave^66,67^. As depicted in Fig. 5c, the SHG intensity with the AuNCs exhibits 37% enhancement compared with the pristine monolayer WS_2_ on the Al_2_O_3_-Au film. Considering the nanocube size and the pumping laser spot (10.45 μm^2^, see Supplementary Information S7 for more details), the 〈EF〉 of SHG in a nanocavity is estimated to be 134. The smaller enhancement factor of SHG than the doubly resonant upconversion can be attributed to the single resonance with the excitation light and the unoptimized collection efficiency (see Supplementary Information Fig. S16 for more details).

Finally, Fig. 5d illustrates the physical scenarios of the SHG, downconversion PL, and TPL, where CB and VB represent the conduction and valence bands, respectively. Specifically, hot carriers are initially generated through above-bandgap photon absorption, followed by relaxation to band edges and subsequent radiative recombination. In nonlinear PL processes, multiphoton absorption creates excited electrons through either degenerate or nondegenerate channels. Those carriers rapidly undergo nonradiative relaxation to excitonic states and then emit photons. Meanwhile, due to the broken inversion symmetry, the SHG can also occur in WS_2_ monolayers^68^.

## Discussion

In summary, the giant TPL enhancement and physical scenario for coupling 2D excitons with doubly resonant plasmonic nanocavity were systematically investigated. Specifically, due to optimized light collection efficiency, enhanced excitation rates, and Purcell-effect-mediated quantum yield improvement, a 2440-fold TPL enhancement was obtained. Additionally, exciton energy dependent upconversion for the evolution of the coupled system was investigated, demonstrating the optimized enhancement factor >3000 at 350 K and the thermally tuned excitonic upconversion. Finally, the single resonance enhanced PL (~890-fold) and SHG (~134-fold) were elaborately demonstrated, deepening our understanding on the giant TPL enhancement by the doubly resonant plasmonic cavity. These results establish a foundation for developing cost-effective, high-performance nonlinear photonic devices and probing fine excitonic states via configuring plasmonic nanocavities.

## Materials and methods

### Sample preparations

A 50 nm Au film followed by a 7 nm Al_2_O_3_ dielectric layer was sequentially deposited onto Si wafers carrying 300 nm SiO_2_ by electron beam evaporation (DE400). The Al_2_O_3_ barrier originates from the complete oxidation of an Al layer, where the Al thickness is monitored by an integrated crystal oscillator sensor (SQS-242) and converted into a thickness of 7.0 ± 0.1 nm via the system’s built-in algorithm. Then the mechanically exfoliated WS_2_ monolayer was transferred onto the substrate assisted by the polydimethylsiloxane (PDMS). Subsequently, AuNCs (~170 nm) were uniformly dispersed on the monolayer by a chemical-assisted method. As a standard protocol, PAH solution was prepared by dissolving 58.6 mg NaCl and 45.1 mg PAH in 1 mL deionized (DI) water, whereas PSS solution was obtained by dissolving 58.6 mg NaCl with 212.3 mg PSS in 1 mL DI water. The substrate was carefully cleaned with DI water at room temperature by being immersed in PAH and PSS solutions alternately, and held for 5 min after each immersion. During this stage, an organic adhesive layer was assembled onto the substrate through layer-by-layer assembly process^69,70^. The resultant layer exhibited a thickness of ~1 nm. Finally, a solution of AuNCs (50 mg l⁻¹ in DI water) was delivered onto the substrate at a dosage of ~100 µl cm⁻² using a pipette gun. The substrate was then dried on a 90 °C hot plate in air.

### Dark-field scattering measurements

Dark-field scattering mapping and spectroscopic measurement were conducted on a commercial hyperspectral imaging system (Cytoviva, HISV3). Illumination was delivered through a 100× objective (Olympus, MPlanFLN, NA = 0.9) that tightly focused a broadband white-light beam. The scattering signal mapping was achieved with a high-precision motorized stage, enabling the acquisition of spectral profiles for every pixel. The Scattering spectra were detected by a spectrometer (Horiba, iHR550) cooled to −60 °C. All scattering signals were subsequently corrected against the substrate using the integrated software (Cytoviva ENVI 4.8).

### Confocal PL spectrum measurements

PL spectra mappings were performed by a confocal μ-Raman spectroscopy system (XperRam200, Nanobase, South Korea). A continuous laser beam (532 nm, 0.2 mW) is focused onto the sample by a microscope objective (40×, NA = 0.75).

### AFM morphology characterization

Surface morphology of the sample was measured by a Bruker Dimension Icon atomic force microscope equipped with a NanoScope 6 AFM controller. All measurements were performed under ambient conditions with careful alignment and calibration to guarantee both measurement accuracy and data reproducibility.

### Mirco-PL spectra measurements

Thermal control was achieved by the LINKAM THMS600 Heating & Freezing Microscope Stage, which spans −193–697 °C with a precision of ±0.1 °C. The mode-locked Ti:sapphire oscillator (Coherent Mira 900) delivered femtosecond pulses (800 nm, 100 fs, 80 MHz); these pulses were focused through an infinity-corrected, long-working-distance objective (Mitutoyo 100×, NA = 0.5) on the sample placed in the LINKAM stage for optical excitation. Spectroscopic acquisition was performed with a 700 nm short-pass edge filter (Thorlabs FELH0700) to block residual excitation light; the filtered emission was then delivered to the Andor Kymera-328i-A spectrometer (spectral resolution 0.4 nm) equipped with an Andor Newton DU971P EMCCD. The conventional photoluminescence was excited by SHG via BBO crystal from the fundamental waves (800 nm, 100 fs, 80 MHz). The 400 nm pulses were then focused through an infinity-corrected, long-working-distance objective (Mitutoyo 100×, NA = 0.5) for optical excitation. After removed the excitation light through the 500 nm long-pass edge filter (Thorlabs FELH0500), the remaining signal was subsequently directed into the above Andor spectrometer for detection.

### Fluorescence lifetime measurements

The fluorescence lifetime was measured by the FastFLIM system excited by a 405 nm pulsed laser (repetition frequency 40 MHz). The PL signal filtered through a 480 nm long-pass edge filter was collected by Nikon TE2000 microscope with a 60× NA = 1.2 WI objective lens. The range of fluorescence lifetime measurements for the FastFLIM system is 100 ps ~ 100 ms, with a minimum time resolution of ≤1 ps.

### Finite-difference time-domain (FDTD) simulations

3D-FDTD simulations were employed to simulate electromagnetic field properties. In the simulation, the relative permittivities of Si, SiO_2_, Al_2_O_3_, Au, and WS_2_ monolayers were taken from the literature^71–73^, AuNCs with an average edge length of 170 nm was set and the thickness of the SiO_2_, Al_2_O_3_, Au film and WS_2_ layer was set as 300 nm, 7 nm, 50 nm and 0.9 nm, respectively. The mesh size was set to 1 nm around the plasmonic cavity, 0.1 nm in the vicinity of the monolayer (with appropriate extension), and no more than 30 nm in other regions. The total-field scattered-field source with wavelengths ranging from 500 to 950 nm was used. The in-plane dipole source arrays with a center wavelength of 620 nm and spectral width of 25 nm were used to simulate the far-field angular radiation patterns. Plane monitors were placed above the gold nanoparticles and below the gold film within the single homogeneous medium. The perfectly matched layer was set as the boundary conditions.

## Supplementary information

Supplementary Information

## Data Availability

The data supporting these findings could be found in the Supplementary Information file published along this paper.
