# Supplementary material for: Giant two-photon upconversion from 2D exciton in doubly-resonant plasmonic nanocavity
Source: Light Sci Appl. 2025 Sep 10;14:312. doi: 10.1038/s41377-025-02010-w (PMC12423298; doi:10.1038/s41377-025-02010-w)
Supplement: Supplementary file 1 — Supplementary Information [file 41377_2025_2010_MOESM1_ESM.docx]

**Supplementary Information for**

**Giant two-photon upconversion from 2D exciton in doubly-resonant plasmonic nanocavity**

*Fangxun Liu,^1, #^ Haiyi Liu,^1, #^ Cheng Chi,^2, #^ Wenqi Qian,^1^ Yuchen Dai,^3^ Guangyi Tao,^3^ Sihan Lin,^1^ Shihan Ding,^1^ Menghan Yu,^1^ Hongliang Liu,^1^ Lie Lin,^1^ Pengfei Qi,^1, 4, *^ Zheyu Fang^3^ and Weiwei Liu^1^*

*^1^Institute of Modern Optics, Nankai University, Tianjin Key Laboratory of Micro-scale Optical Information Science and Technology, Tianjin 300350, China*

*^2^Beijing Engineering Research Center of Mixed Reality and Advanced Display, School of Optics and Photonics, Beijing Institute of Technology, Beijing 100081, China*

*^3^School of Physics, State Key Laboratory for Mesoscopic Physics, Academy for Advanced Interdisciplinary Studies, Collaborative Innovation Center of Quantum Matter, Nano-optoelectronics Frontier Center of Ministry of Education, Peking University, Beijing 100871, China*

*^4^Academy for Advanced Interdisciplinary Studies, Nankai University, Tianjin 300350, China*

^*^*Email:* [*qipengfei@nankai.edu.cn*](mailto:qipengfei@nankai.edu.cn)

*^#^These authors contributed equally:* *Fangxun Liu, Haiyi Liu and Cheng Chi*

CONTENTS

[S1. Atomic force microscopy topography image 1](#_Toc205413190)

[S2. Normalized PL and Raman spectrum 1](#_Toc205413191)

[S3. Theoretical Modeling 2](#_Toc205413192)

[S4. Unprocessed data of Figure 1f 2](#_Toc205413193)

[S5. Power-dependent PL spectra of monolayer WS_2_ 3](#_Toc205413194)

[S6. Plasmonic-nanocavity-enhanced upconverted emission 4](#_Toc205413195)

[S7. Spot Size of excitation laser 5](#_Toc205413196)

[S8. Normalized time-resolved luminescence decay 6](#_Toc205413197)

[S9. Schematics for simulating far-field radiation pattern 6](#_Toc205413198)

[S10. Far-field radiation for monolayer WS_2_ in free space and plasmonic cavity 7](#_Toc205413199)

[S11. Schematic diagram of AuNCs-substrate interaction 8](#_Toc205413200)

[S12. Magnetic field distributions around plasmonic cavity 9](#_Toc205413201)

[S13. Temperature-dependent normalized PL spectra 10](#_Toc205413202)

[S14. Power-dependent DC-PL for WS_2_ monolayers 10](#_Toc205413203)

[S15. Power-dependent SHG for WS_2_ monolayers 11](#_Toc205413204)

[S16. SHG far-field angular radiation patterns in the plasmonic cavity 11](#_Toc205413205)

1. Atomic force microscopy topography image


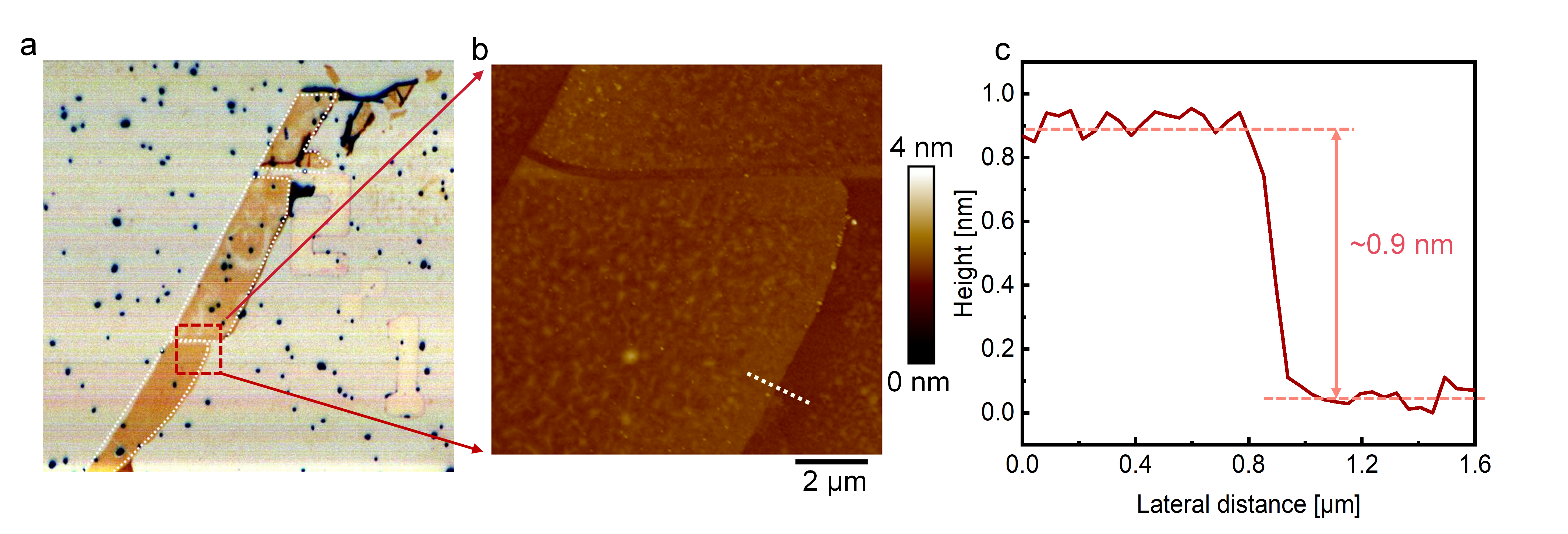


**Figure S1. Atomic force microscopy topography image of the monolayer WS_2_.** (a) Optical microscopy image of the monolayer WS_2_. (b) Atomic force microscopy image of the region within the red dotted box in (a). (c) Height profile along white dashed line in (b), indicating monolayer WS_2_ with the thickness of ~0.9 nm.

1. Normalized PL and Raman spectrum


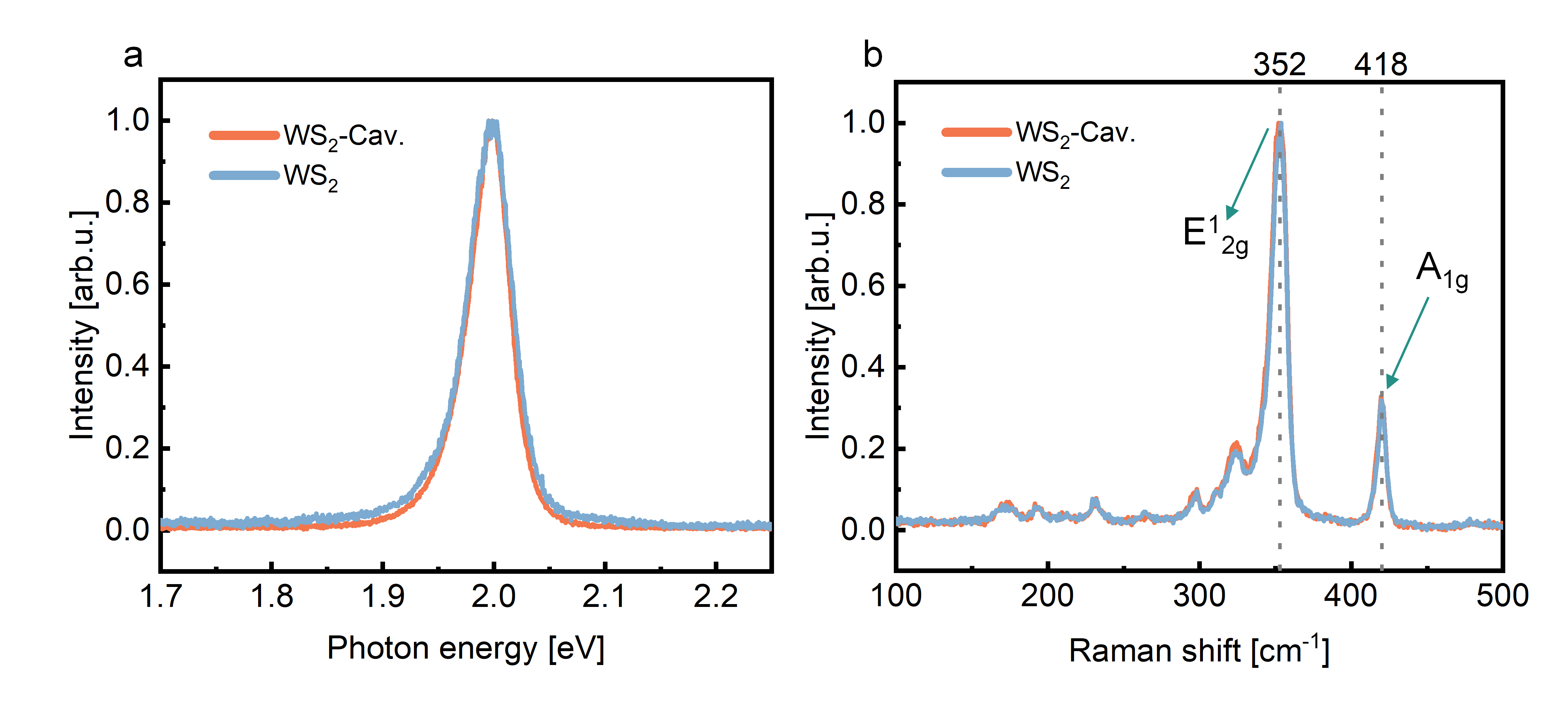


**Figure S2. Normalized PL and Raman spectrum.** (a) Comparison of the normalized PL spectrum for WS_2_ deposited on the Au film (blue) and in the nanocavity (red). (b) Comparison of the normalized Raman spectrum for WS_2_ deposited on the Au film (blue) and in the nanocavity (red).

1. Theoretical Modeling


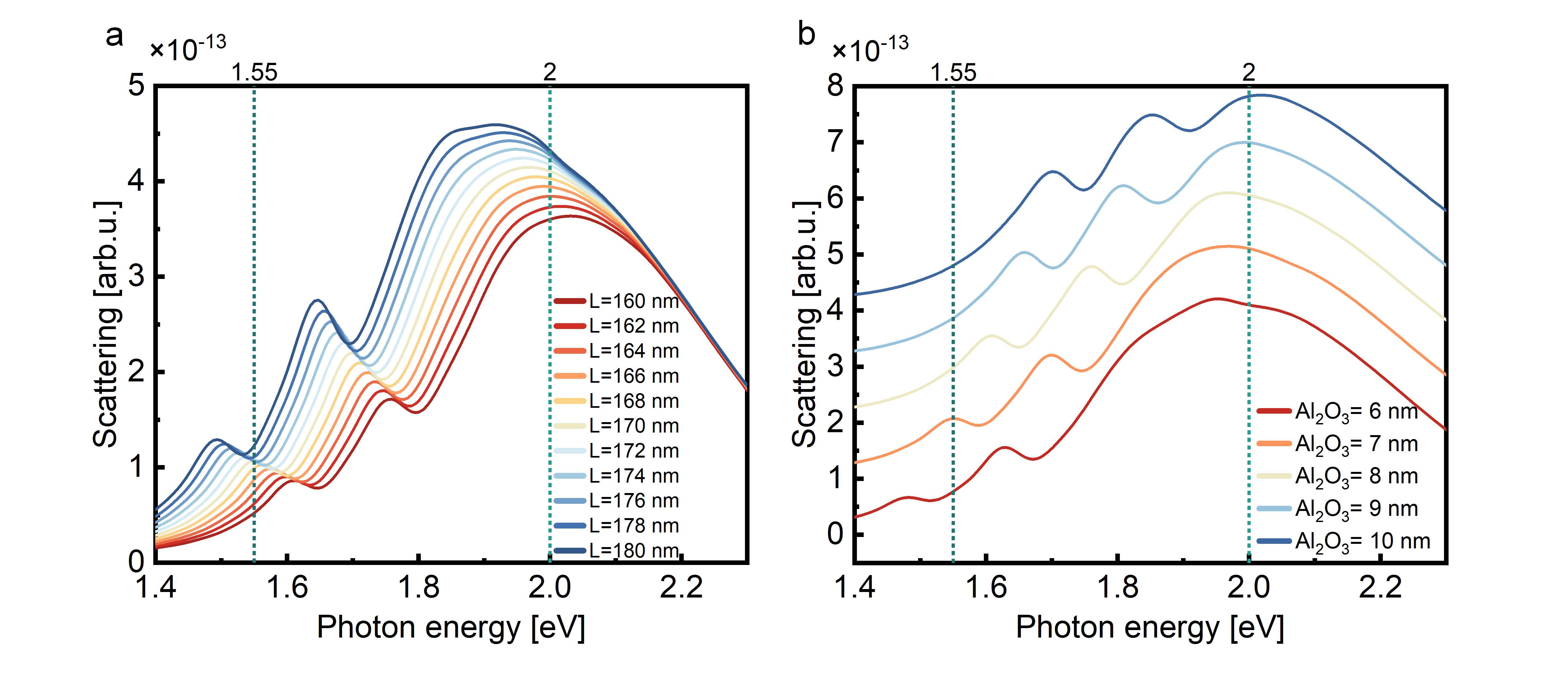


**Figure S3.** **Simulated** **scattering spectra.** (a) Simulated scattering spectra of hybrid nanostructures with gold nanocubes of various sizes (160-180 nm in cube edge length). (b) Simulated scattering spectra of plasmonic nanocavities with various thickness of spacer Al_2_O_3_.

1. Unprocessed data of Figure 1f


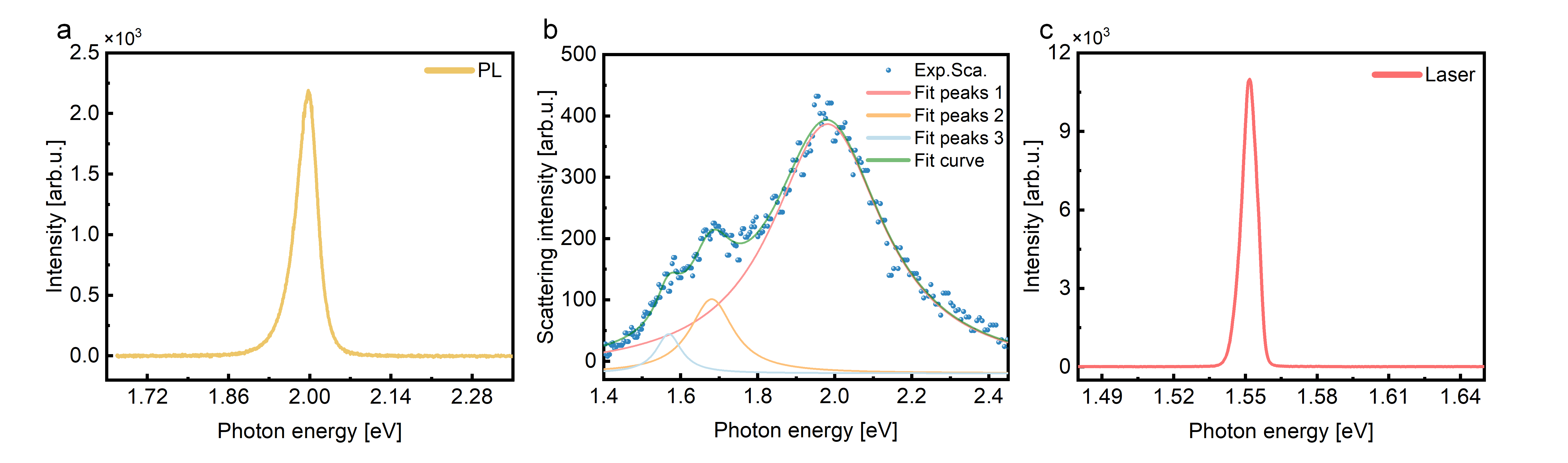


**Figure S4.** **Unprocessed data of Figure 1f**. (a) PL spectra of monolayer WS_2_. (b) Experimental scattering spectrum of a plasmonic nanocavity. (c) Excitation laser.

1. Power-dependent PL spectra of monolayer WS_2_


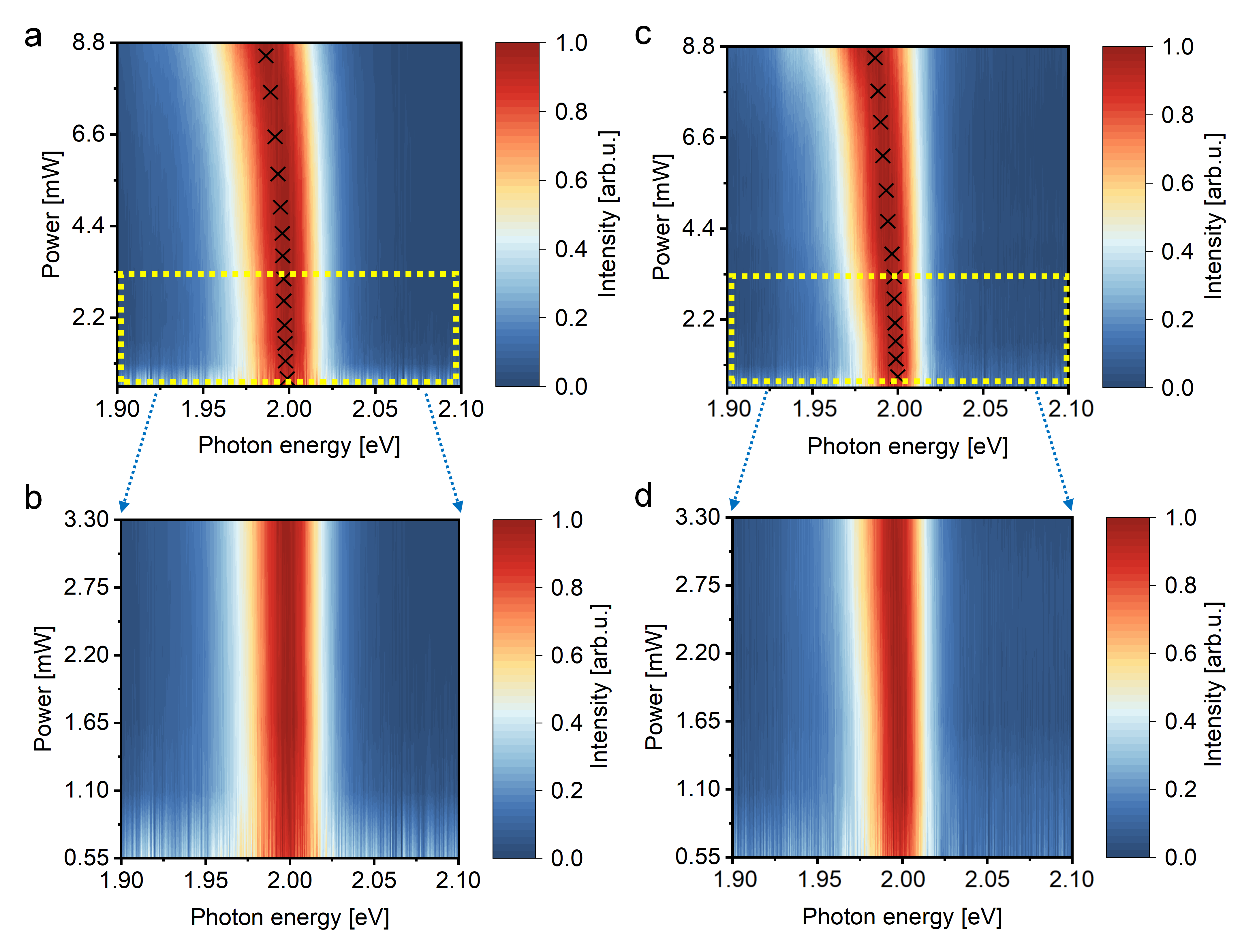


**Figure S5**. **Power-dependent PL spectra of monolayer WS_2_.** (a) Power-dependent (0.55 mW to 8.8 mW) PL spectra of monolayer WS_2_ on the Au film. (b) Power-dependent (0.55 mW to 3.3 mW) PL spectra of monolayer WS_2_ on the Au film. (c) Power-dependent (0.55 mW to 8.8 mW) PL spectra of monolayer WS_2_ in plasmonic cavity. (d) Power-dependent (0.55 mW to 3.3 mW) PL spectra of monolayer WS_2_ in plasmonic cavity.

1. Plasmonic-nanocavity-enhanced upconverted emission


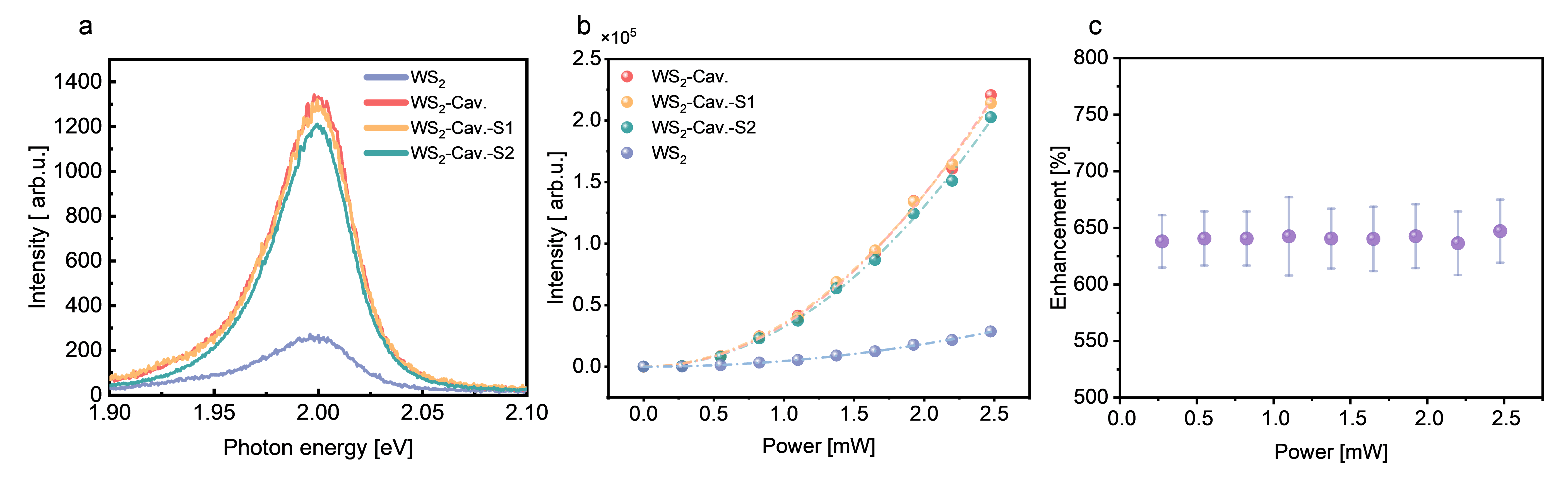


**Figure S6.** **Plasmonic-nanocavity-enhanced upconverted emission in other plasmonic nanocavities** (a) Enhanced upconverted emission spectra of supplementary plasmonic nanocavities S1 and S2 at the condition of Figure 2c. (b) Excitation power-dependent integrated upconverted emission intensity for supplementary plasmonic nanocavities S1 and S2. (c) Statistical metrics on enhancement variability.

1. Spot Size of excitation laser





**Figure S7.** **Spot Size of excitation laser.** Spatial intensity profiles of the (a) 400 nm and (b) 800 nm excitation laser. Inset: micrograph of focused laser beam. Scale bar is 3 μm. The (c) 400 nm and (d) 800 excitation laser spot radiuses using the knife-edge technique.

1. Normalized time-resolved luminescence decay


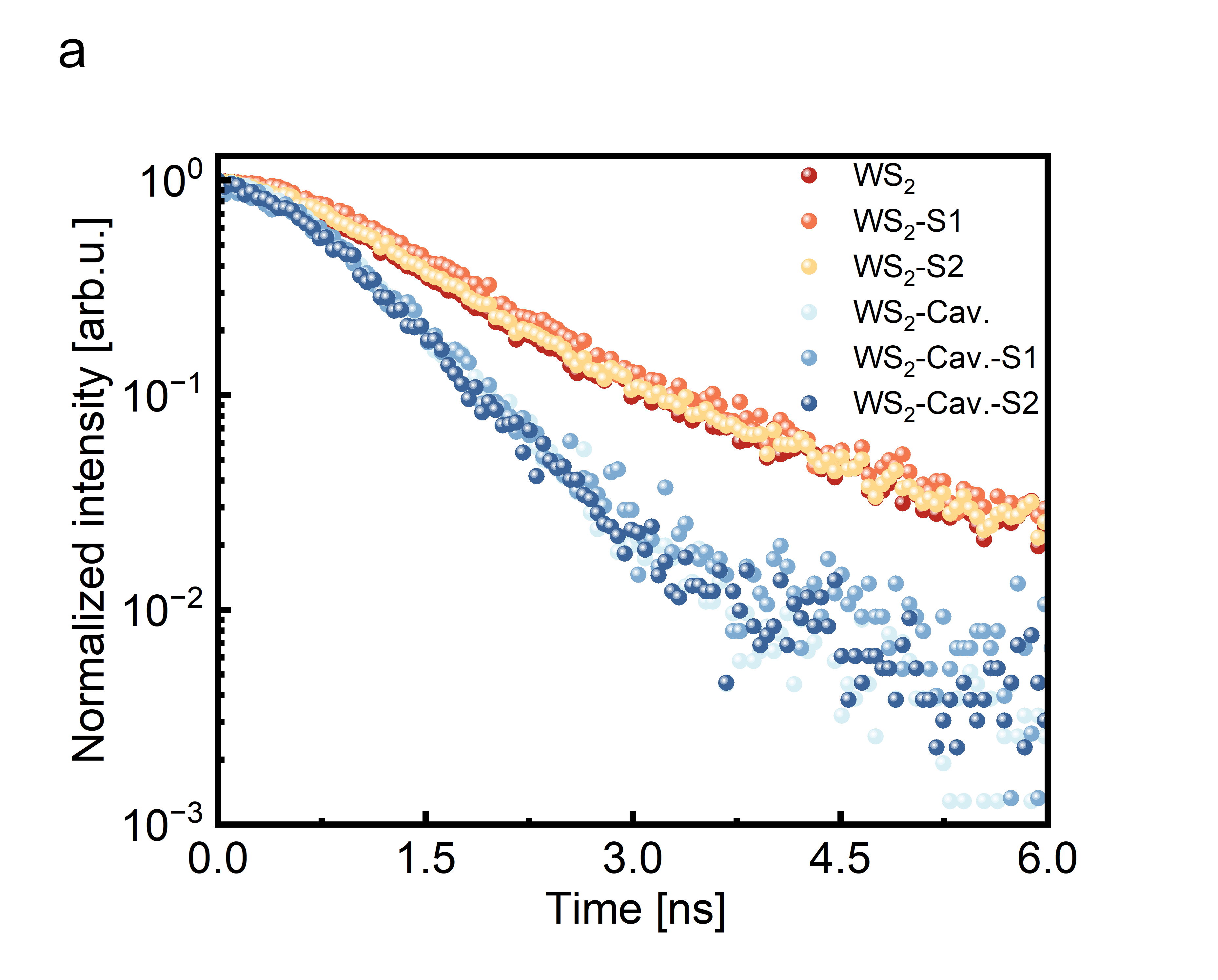


**Figure S8.** Normalized time-resolved luminescence decay for WS_2_ deposited on the Au film and WS_2_ enhanced by the nanocavity mode from different positions of the same sample at an emission wavelength of 620 nm.

1. Schematics for simulating far-field radiation pattern


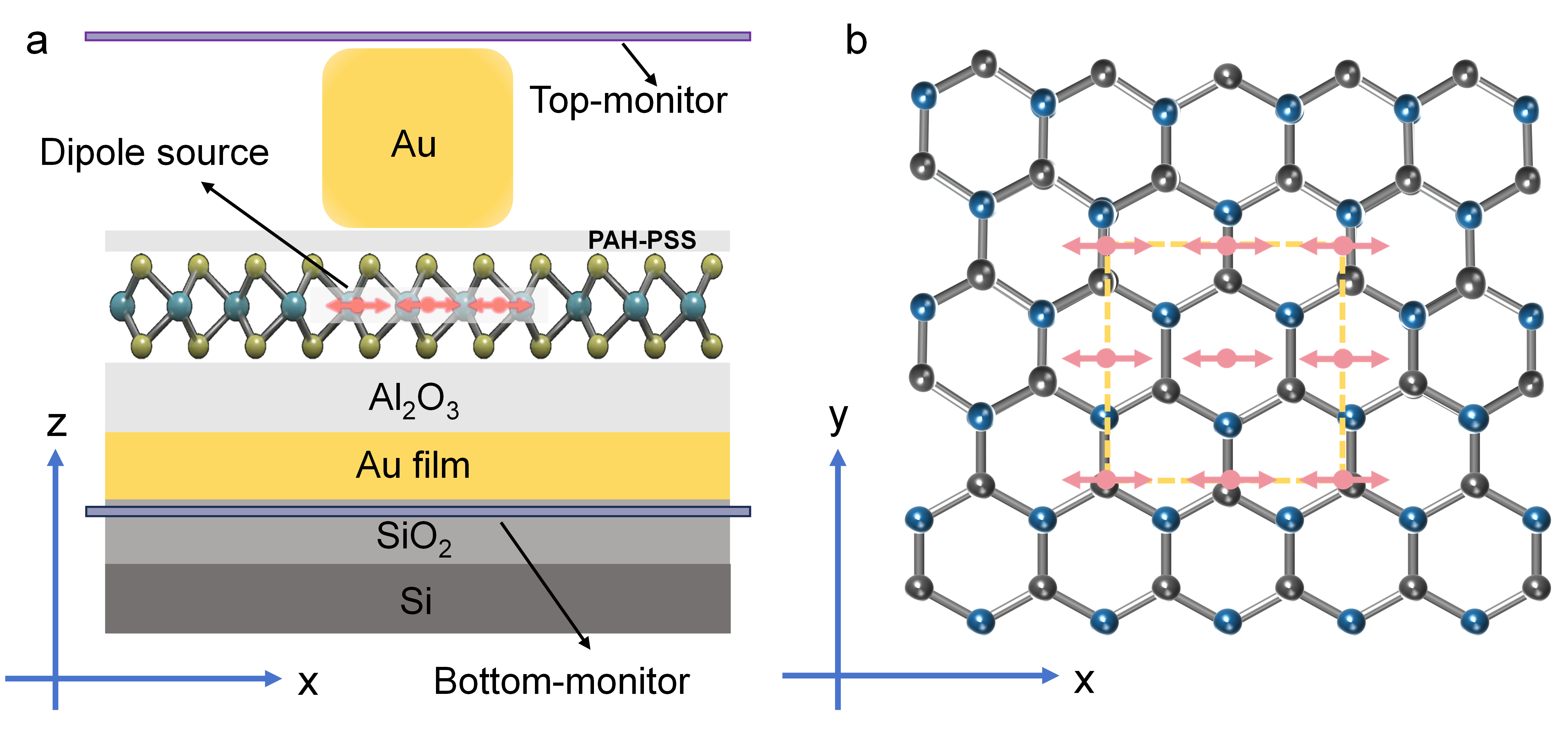


**Figure S9.** **Schematics for simulating far-field radiation pattern.** The in-plane dipole source arrays with center wavelength of 620 nm and spectral width of 25 nm were adopted.

1. Far-field radiation for monolayer WS_2_ in free space and plasmonic cavity


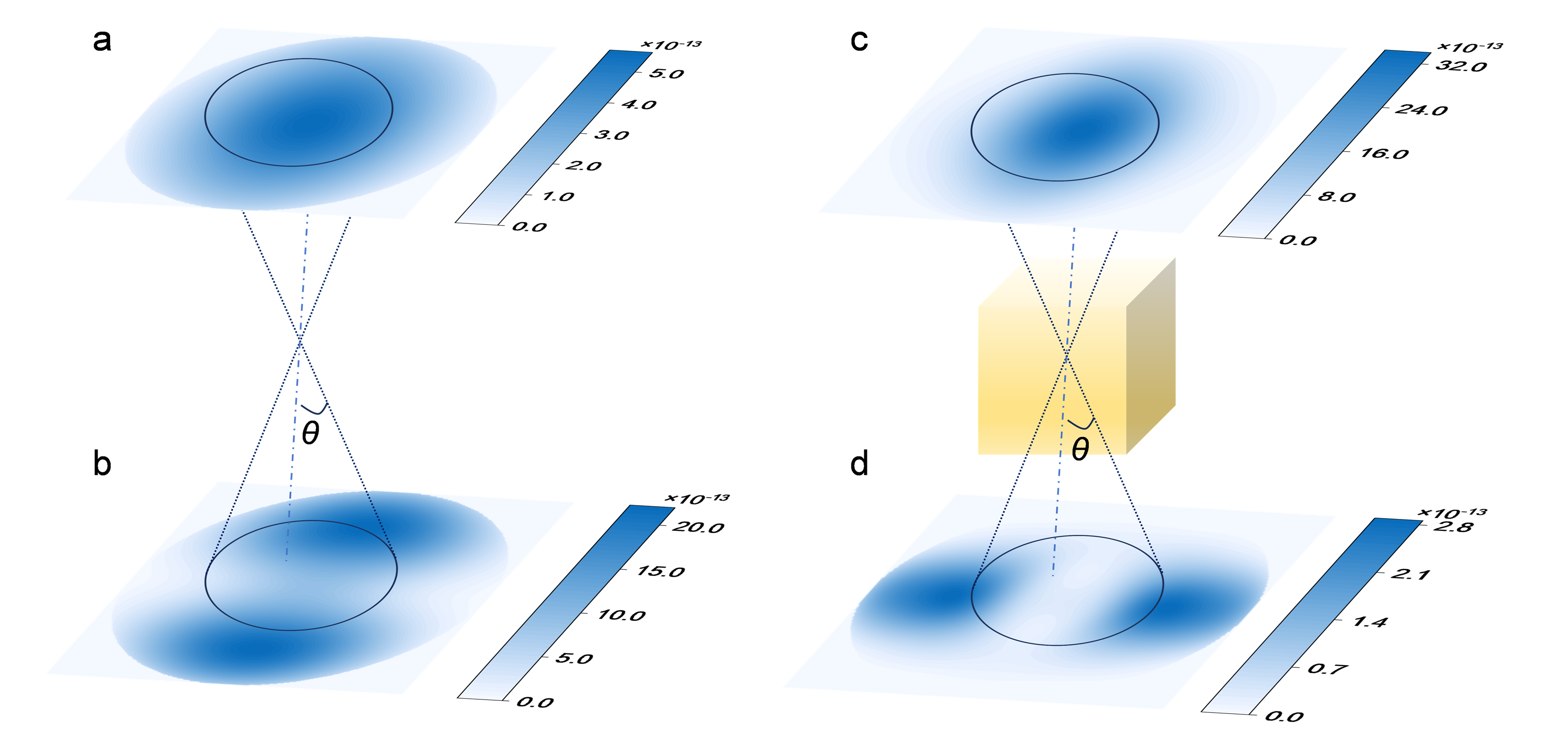


**Figure S10. Far-field radiation patterns for monolayer WS_2_ in free space and plasmonic cavity.** (a), (b) Far-field radiation patterns for a monitor placed on the top (a) and bottom (b) of the monolayer WS_2_. (c), (d) Far-field radiation patterns for a monitor placed on the top (c) and bottom (d) of the monolayer WS_2_ in plasmonic cavity. The average intensity around the angle *θ* can be calculated and plotted as the far-field angular radiation patterns in Figures 3b and 3c.

1. Schematic diagram of AuNCs-substrate interaction


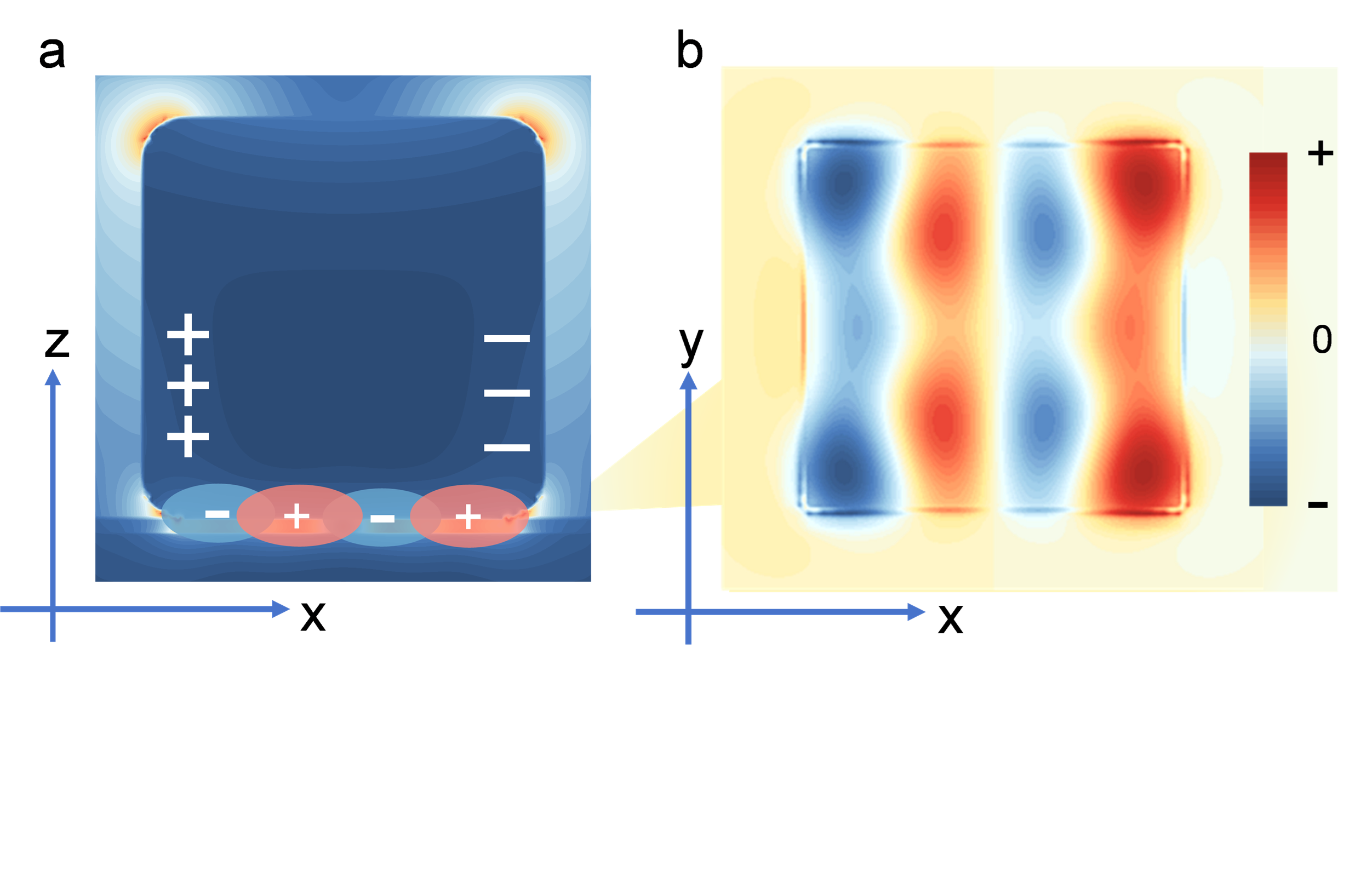


**Figure S11.** **Schematic diagram of AuNCs-substrate interaction.** (a) Schematic diagram of AuNCs-substrate interaction for the cavity mode at 2.0 eV. (b) Charge distribution at the monolayer WS_2_ for the cavity mode at 2.0 eV.

As shown in Figures S11a and S11b, the image dipole will form in substrate and couple with the SPR of Au nanocubes, leading to strong field enhancement. The fields shown in Figures 3f and 3i have both in-plane and out-plane electric field components. Therefore, the confined electromagnetic field can interact with the 2D exciton with in-plane dipole.

1. Magnetic field distributions around plasmonic cavity


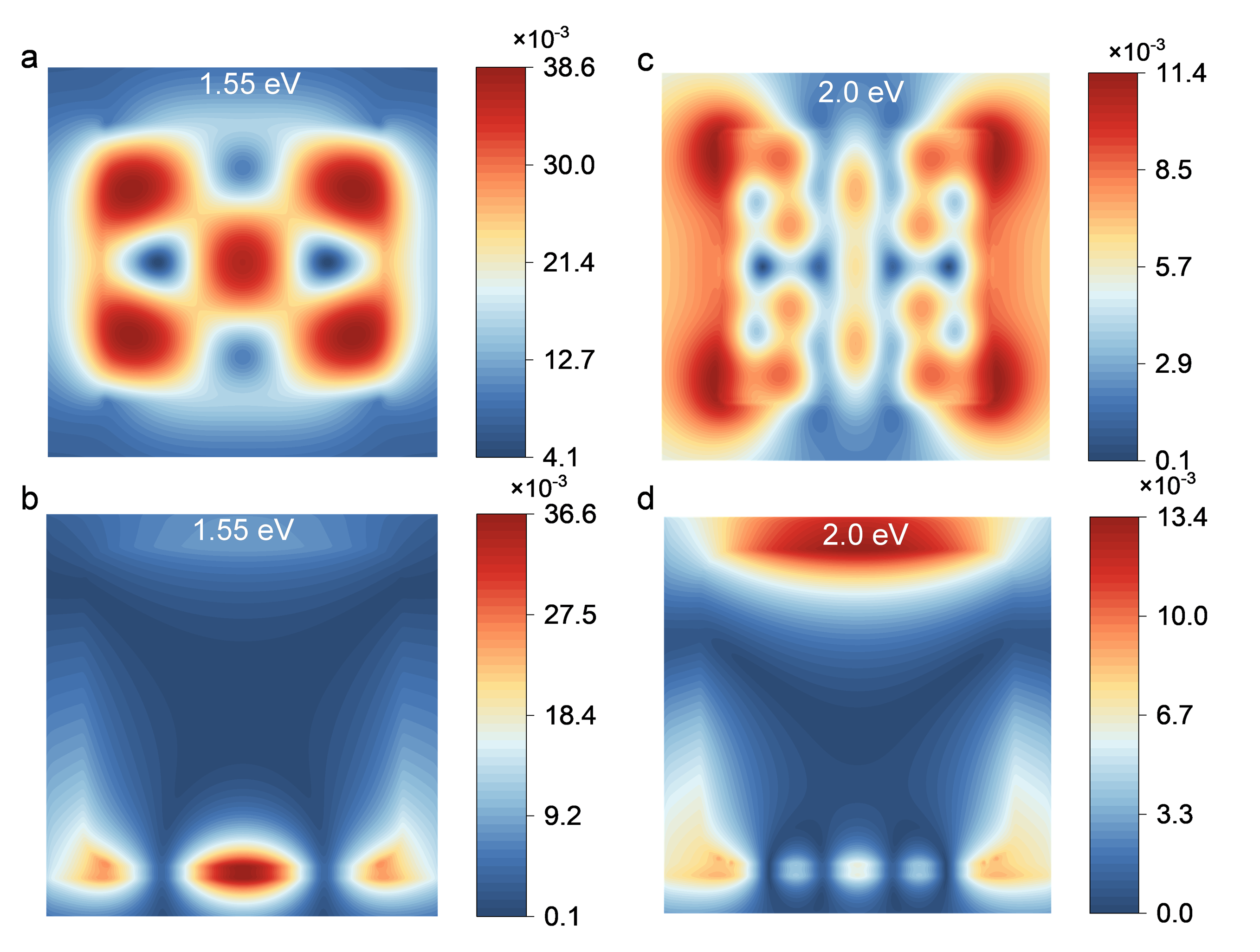


**Figure S12. Magnetic field distributions around plasmonic cavity.** (a), (b) Magnetic field distributions around plasmonic cavity at 1.55 eV. (c), (d) Magnetic field distributions around plasmonic cavity at 2.0 eV. (a), (c) corresponds to the magnetic field distributions at the monolayer WS_2_ plane, and (b), (d) corresponds to the magnetic field distributions at the xz plane.

1. Temperature-dependent normalized PL spectra


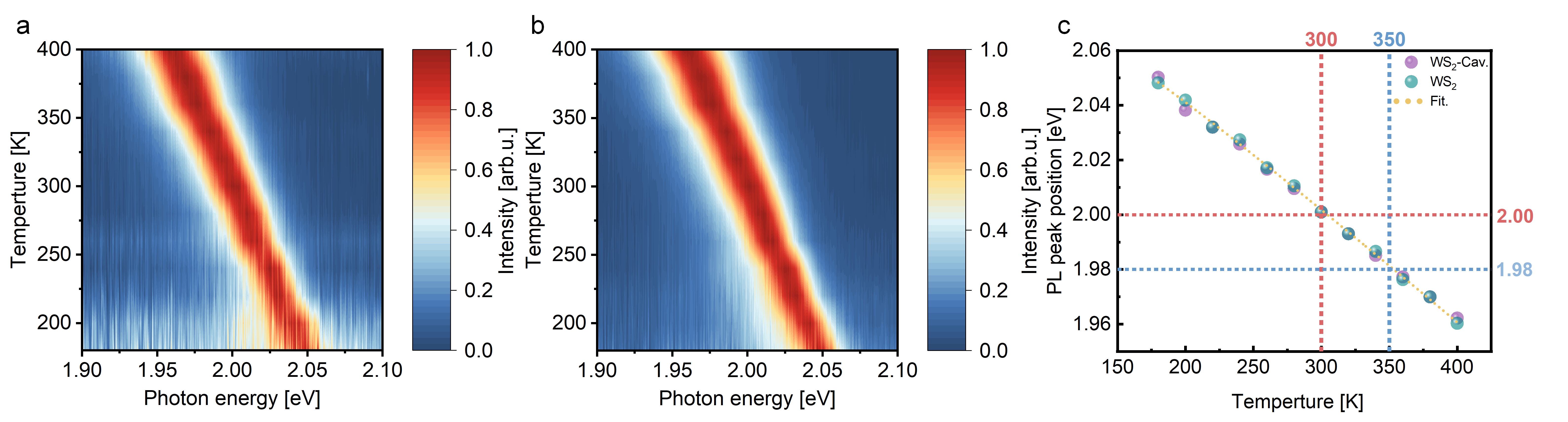


**Figure S13. Temperature-dependent normalized PL spectra and peak position.** (a)Temperature-dependent normalized PL spectra for WS_2_ monolayers on Au/SiO_2_/Si. (b)Temperature-dependent normalized PL spectra for WS_2_ monolayers in the plasmonic cavity. (c)Comparison of temperature-dependent exciton peak positions between WS_2_ monolayers on Au/SiO_2_/Si and in plasmonic cavities.

1. Power-dependent DC-PL for WS_2_ monolayers


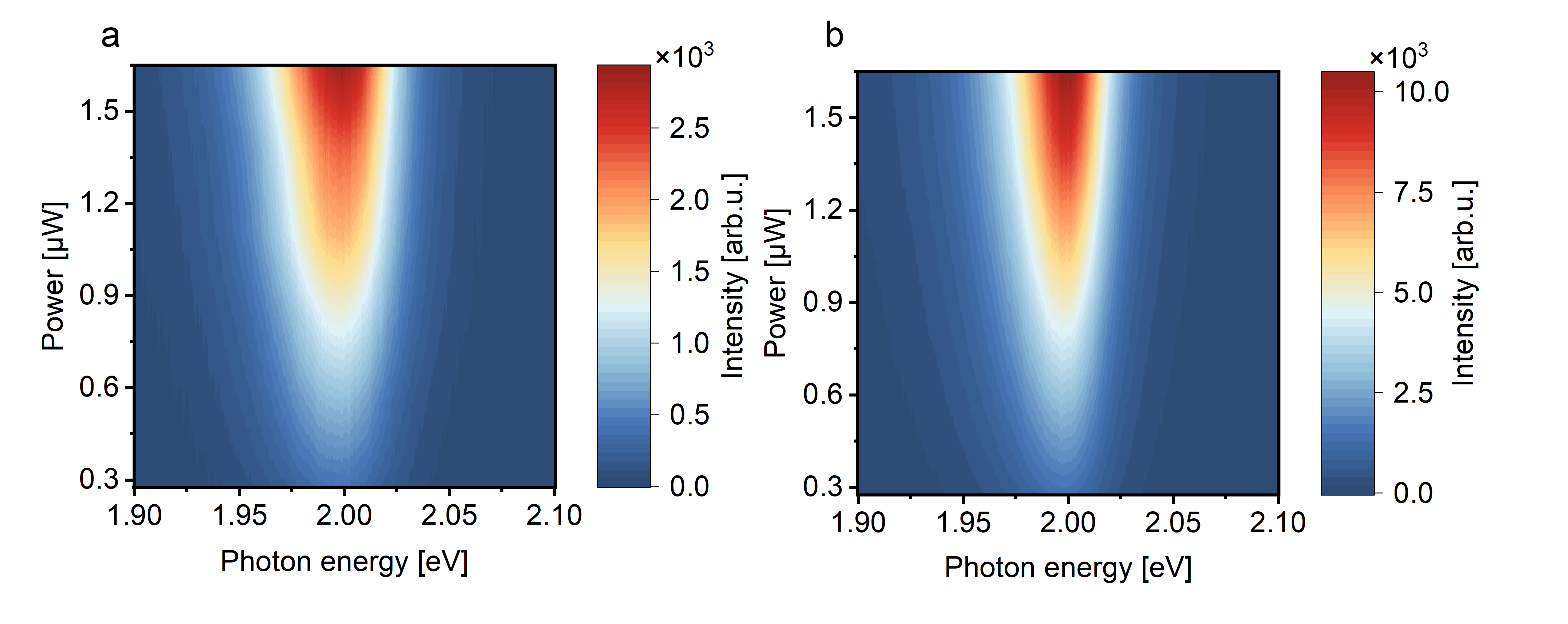


**Figure S14. Power-dependent DC-PL for WS_2_ monolayers.** Excitation-power-dependent DC-PL spectra for WS_2_ monolayers on Au/SiO_2_/Si (a) and in the plasmonic cavity (b).

1. Power-dependent SHG for WS_2_ monolayers


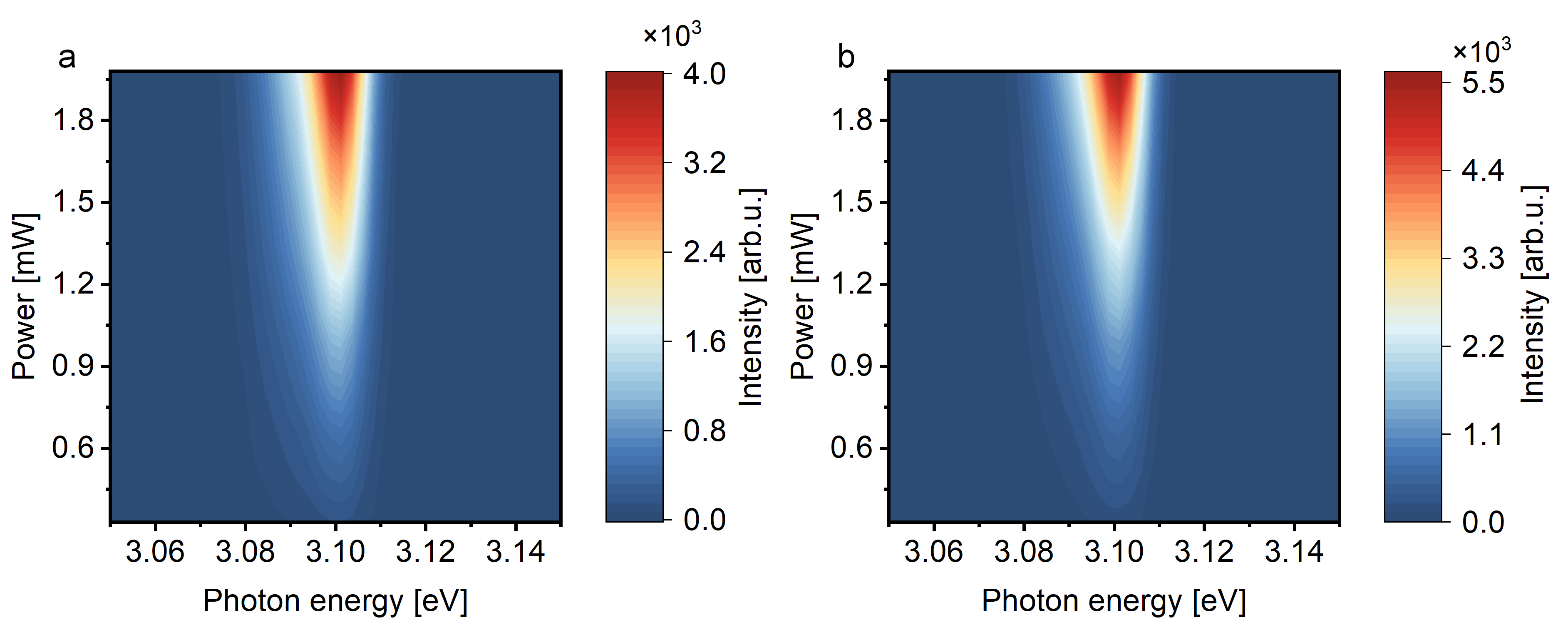


**Figure S15.** **Power-dependent SHG for WS_2_ monolayers.** Excitation-power-dependent SHG for WS_2_ monolayers on Au/SiO_2_/Si (a) and in the plasmonic cavity (b). Here the SHG in WS_2_ monolayers can be confirmed by two pieces of evidences: (1) The photon energy of excitation pulse is 1.55 eV, the emitted photon energy is about 3.10 eV; (2) As depicted in figure 5c, the dependence of the SHG intensity on the excitation power for monolayers can be fitted well by the power law formula *I* = b*P*^2^.

1. SHG far-field angular radiation patterns in the plasmonic cavity


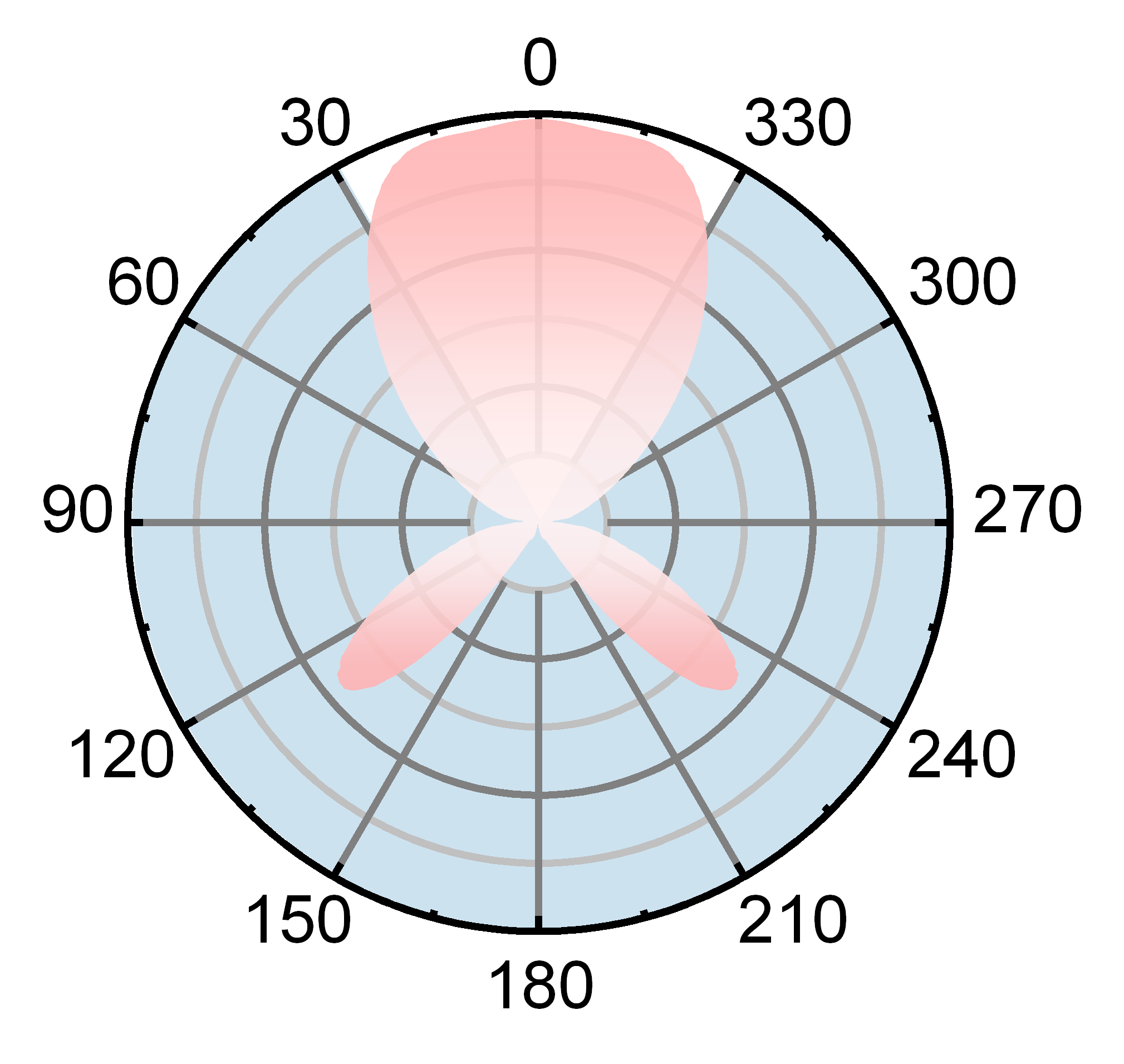


**Figure S16. SHG far-field angular radiation patterns for monolayer WS_2_ in the plasmonic cavity.** The fraction of emitted light collected by the objective lens with NA = 0.5 used in our measurements can be calculated to be 43.69%.
